# Supplementary material for: A physical model for M1-mediated influenza A virus assembly
Source: Biophys J. 2024 Nov 20;124(1):134–44. doi: 10.1016/j.bpj.2024.11.016 (PMC11739876; doi:10.1016/j.bpj.2024.11.016)
Supplement: Document S1. Figure S1 [file mmc1.pdf]

Biophysical Journal, Volume *124*

Supplemental information

A physical model for M1-mediated influenza A virus assembly

Julia Peukes, Serge Dmitrieff, François J. Nédélec, and John A.G. Briggs

Supplemental data

Figure S1

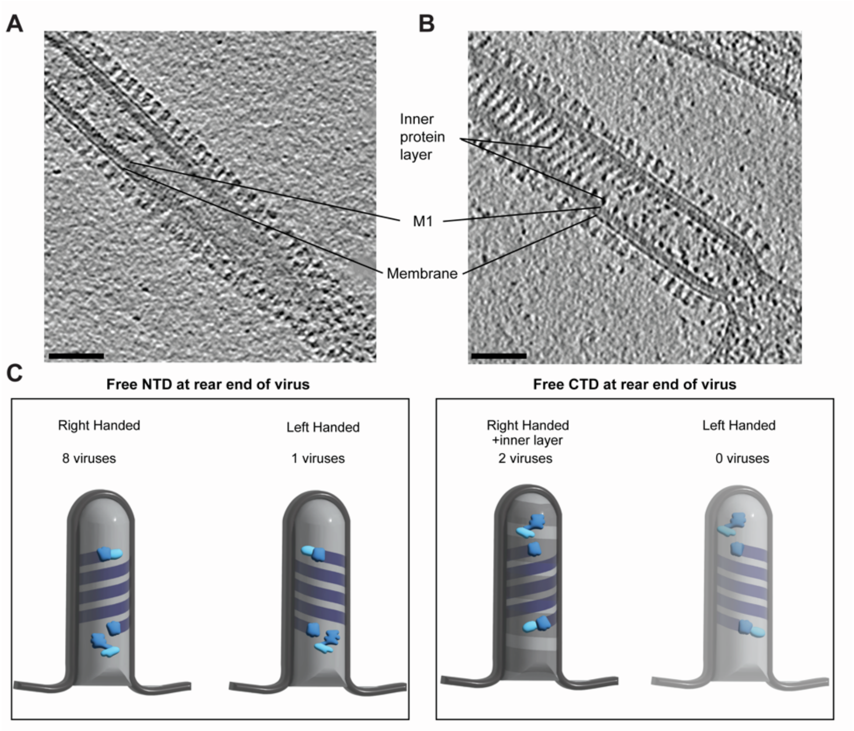

M1 orientation for M1 strands with different handedness and in the presence of an additional inner protein layer. A) Slice through a representative tomogram of an influenza A virus filament without an additional inner protein layer. B) Slice though a tomogram of an influenza A virus containing an additional protein layer at the inside of M1, which was present in 20 % of viruses. C) Illustrations of M1 orientation relative to M1 handedness and the presence or absence of an additional inner protein layer. Scale bars: 50 nm.
